# Key stakeholder perceptions about consent to participate in acute illness research: a rapid, systematic review to inform epi/pandemic research preparedness

**DOI:** 10.1186/s13063-015-1110-6

**Published:** 2015-12-29

**Authors:** Nina H. Gobat, Micaela Gal, Nick A. Francis, Kerenza Hood, Angela Watkins, Jill Turner, Ronald Moore, Steve A. R. Webb, Christopher C. Butler, Alistair Nichol

**Affiliations:** Cochrane Institute of Primary Care and Public Health, Cardiff University, Neaudd Meirionnydd, Heath Park Campus, Cardiff, Wales CF14 4YS UK; College of Biomedical and Life Sciences, Cardiff University, Cardiff, Wales UK; University College Dublin, Dublin, Ireland; University of Western Australia, Perth, Australia; Oxford University, Oxford, UK

## Abstract

**Background:**

A rigorous research response is required to inform clinical and public health decision-making during an epi/pandemic. However, the ethical conduct of such research, which often involves critically ill patients, may be complicated by the diminished capacity to consent and an imperative to initiate trial therapies within short time frames. Alternative approaches to taking prospective informed consent may therefore be used. We aimed to rapidly review evidence on key stakeholder (patients, their proxy decision-makers, clinicians and regulators) views concerning the acceptability of various approaches for obtaining consent relevant to pandemic-related acute illness research.

**Methods:**

We conducted a rapid evidence review, using the Internet, database and hand-searching for English language empirical publications from 1996 to 2014 on stakeholder opinions of consent models (prospective informed, third-party, deferred, or waived) used in acute illness research. We excluded research on consent to treatment, screening, or other such procedures, non-emergency research and secondary studies. Papers were categorised, and data summarised using narrative synthesis.

**Results:**

We screened 689 citations, reviewed 104 full-text articles and included 52. Just one paper related specifically to pandemic research. In other emergency research contexts potential research participants, clinicians and research staff found third-party, deferred, and waived consent to be acceptable as a means to feasibly conduct such research. Acceptability to potential participants was motivated by altruism, trust in the medical community, and perceived value in medical research and decreased as the perceived risks associated with participation increased. Discrepancies were observed in the acceptability of the concept and application or experience of alternative consent models. Patients accepted clinicians acting as proxy-decision makers, with preference for two decision makers as invasiveness of interventions increased. Research regulators were more cautious when approving studies conducted with alternative consent models; however, their views were generally under-represented.

**Conclusions:**

Third-party, deferred, and waived consent models are broadly acceptable to potential participants, clinicians and/or researchers for emergency research. Further consultation with key stakeholders, particularly with regulators, and studies focused specifically on epi/pandemic research, are required. We highlight gaps and recommendations to inform set-up and protocol development for pandemic research and institutional review board processes.

**PROSPERO protocol registration number:**

CRD42014014000

**Electronic supplementary material:**

The online version of this article (doi:10.1186/s13063-015-1110-6) contains supplementary material, which is available to authorized users.

## Background

Infectious disease pandemics are recurrent but unpredictable events that have a significant impact on the health, economy and security of societies worldwide [1]. Emerging infections that lead to epidemic or pandemic outbreaks arise at the human-animal interface [2]. The amplification and spread of these diseases can result in outbreaks and epidemics that may develop into a public health emergency. A pandemic occurs when there is global spread of the disease [1]. The World Health Organisation (WHO) monitors and reports pandemics in terms of global phases – inter-pandemic, alert, pandemic and transition [3]. These phases are designed to inform national pandemic risk management strategies and actions. Through all phases, expedient, high-quality epidemiological and clinical research is essential to inform clinical and public health decision-making [4]. Such research has the potential to shift the trajectory of a pandemic [5, 6]. The need to develop research preparedness alongside clinical and public health response preparedness has been recognised increasingly. Some progress has been made in strengthening surveillance systems and in the development and testing of new vaccines. However, the experiences of attempting to conduct research during recent epi/pandemics, such as the 2009 H1N1 influenza pandemic and the 2014 outbreak of Ebola in West Africa, indicate that a timely and effective research response is often not possible [7].

Hospitals and critical care units, in particular, are at the front line of caring for the most severely ill patients and act as the canary in the coalmine during an epi/pandemic in the initial phase of an outbreak [6, 8, 9]. They are also at the forefront of generating important new knowledge about incidence, outcome, infection control, case presentation, resource utilisation and optimal clinical care and are well placed to provide crucial information to inform both clinical and public health decision-making [10]. Consequently, there is a need to develop both clinical and research pandemic preparedness in critical care units [10]. Clinical research conducted during a pandemic should be held to the same high standards of scientific and ethical rigour as that conducted during non-emergent times. Legislative and moral codes of practice [11] set out the ethical requirements for research, which include that it has value in advancing health or knowledge, that is it methodologically sound and scientifically valid, that the benefit to the individual and society outweighs the risks, and that research participants provide informed consent [12]. Most hospital-based research is subject to review and approval by an independent regulatory body. During the H1N1 pandemic, clinical research was hampered by delays in obtaining these ethical and regulatory approvals, as well as by other factors, such as accessing funding and site recruitment. As a result initial pandemic waves had largely passed by the time recruitment for these studies was ready to commence [10, 13]. As a consequence, recommendations have been made for organised and integrated research preparedness for pandemics and epidemics [14]. This includes the need for operational research capacity, during inter-pandemic periods (‘peace-time’) that can be activated rapidly and effectively when the need arises [7, 10, 15]. Inter-pandemic activities to achieve preparedness include the design and pre-approval of study protocols [16] and the establishment of centralised, rapid regulatory approval processes [4, 7].

One of the challenges to conducting clinical research at varying stages of a pandemic is obtaining valid informed consent from participants affected by the pandemic. Consent is central to the principle of respect for patient autonomy and is an integral part of ethical biomedical research [11]. For informed consent to be valid, participants should receive sufficient information about the study, including the risks involved, for them to make an informed choice about participation; they should understand this information; and they should be competent to decide and to make the decision voluntarily, that is, in the absence of coercion. Potential participants should understand that they have a right to refuse as well as to withdraw from a study without fear of any consequences [11]. However, many forms of illness with pandemic potential have clinical consequences that result in diminished capacity to consent for many affected patients. Moreover, for some research questions there is a time imperative for recruitment of individual research participants. These issues create challenges for planning and conducting research during possible future pandemics.

These challenges are not unique to pandemics, but, rather, are generic to studying any form of critical illness that results in the diminished capacity to consent. There are several alternatives to prospective informed consent that allow research to be conducted ethically when participants lack the capacity to provide informed consent. We identified three alternative models in Table 1, namely, third-party consent, deferred consent and waived consent. These alternative consent processes have made it feasible to conduct emergency or critical care research that would not otherwise be possible [17, 18]. Researchers have highlighted the utility of these models when designing protocols for pandemic research [4], and existing pandemic protocols use hybrid models proportionate to the level of pandemic risk and based on an assessment of patient capacity and availability of a surrogate consenter [19, 20].

**Table 1:** Definitions of terms

Emergency research	Research including intensive and critical care research that relates directly to a life-threatening or debilitating condition in which there is a time-imperative for intervention.
Capacity to consent	The person should have the capacity to make a choice about the proposed course of action, knows about the study risks, benefits and alternatives, understands that consent is ‘voluntary and continuing permission’, and understands that consent can be withdrawn at any time.
Prospective informed consent	The decision (written, dated and signed) to take part in a study, which is taken after the person is fully informed about the study nature, its significance, implications and risks. Informed consent can be given by any person capable of giving consent or, where the person is not capable, by a surrogate decision maker. Oral consent in the presence of a witness may be given in exceptional cases.
Third-party consent	Informed consent to research participation is provided by a surrogate or proxy decision maker, for example, a family member or legal representative where the potential participant is unable to provide consent themselves. Proxy consent can also describe the process by which people with the legal right to consent for themselves or as a surrogate can delegate that right to another person.
Deferred consent	When a patient is enrolled into a study, and consent is taken later, either from a surrogate decision maker or from the patient when he/she is able to provide informed consent.
Waiver of consent and Exception from informed consent	A consent procedure that alters elements of informed consent or waives the requirements to obtain informed consent. For example consent may be waived if the research presents no more than minimal risk of harm to subjects and could not be carried out without a waiver.
	Exception from informed consent may also apply for enrolment of participants in emergency research. Here, requirements include consultation with representatives of and public disclosure to the communities in which the study will be conducted prior to study initiation. Deferred consent is still a requirement in most cases.

We review the evidence on acceptability of these different consent models from the perspective of different stakeholders. In the absence of pandemic-specific research, we have looked to emergency research more broadly as it shares many of the features that we might expect in hospital based pandemic research. These features are a lack of participant capacity, sometimes in combination with a research question in which the decision to participate is time-critical. The objectives of this review are to broadly map this terrain, to identify recommendations that are relevant to investigators that plan to develop protocols for pandemic research, and to identify further empirical work that might allow researchers to implement these new procedures in a way that is most acceptable to all stakeholder groups.

## Methods

Rapid review methodology offers a structured and efficient approach to synthesising evidence to inform decision-making [21, 22]. They are conducted in a shorter time frame than full systematic reviews, but retain most of the methodological rigour by using systematic and reproducible methods. Rapid reviews produce similar conclusions to systematic reviews that are sufficient for policy and clinical decision-making [23]. The principles of a rapid review are that decisions taken to expedite the review should be transparent, that the purpose is clearly enunciated, and that potential limitations are acknowledged. To expedite our review we limited our search by year (1996 onward) and language (English language only), 70 % of citations were screened by a second researcher, a single researcher conducted data extraction and quality assessment of each paper, and our analysis involved description and categorisation as opposed to more formal approaches such as meta-summary [22].

### Eligibility criteria

We included empirical research using qualitative, quantitative, or both methods that aimed to report the views of potential research participants, their proxy decision makers, clinicians, or research regulators regarding the different models of consent for participation in emergency research. We included paediatric research but excluded neonatal research due to the unique ethical issues arising in this kind of research [24]. English language publications of research conducted in OECD countries from 1996 onward were included. We excluded studies on consent for elective treatment, end-of-life decisions, vaccinations, screening, genetic testing, organ donation and/or other clinical procedures. Studies reporting on research participation that did not include consent, for example, reports of recruitment or efforts at retention, were also excluded, as were descriptive studies reporting on the consent process without evaluating participant experience or studies on the features of consent documents. Finally, the following types of articles were also excluded: opinion pieces, commentaries, editorials, unpublished dissertations, conference abstracts, book chapters, conference reports, protocol papers and reviews.

### Information sources

We searched the following databases in November 2014: MEDLINE, EMBASE, PsycINFO, Health Management Information Consortium (HMIC) via OvidSP; Science Citation Index Expanded (SCI-EXPANDED), Social Sciences Citation Index (SSCI) via Web of Science SSI; Cochrane Central; and OpenGrey. We also searched WHO publications via their website, and hand-searched the following journals from October 2012–2014: Intensive Care Medicine, Journal of Medical Ethics, BMC Medical Ethics and Critical Care Medicine. Finally, reference lists of included articles and review articles were mined to identify other relevant citations.

### Search strategy

The search strategy was developed using two concepts and synonyms– informed consent and emergency care. In addition, we used an adapted search filter for participant views [25] to enhance the specificity of the search. The full search strategy is available in Additional file 1: Appendix A.

### Study selection

A single researcher (NG) reviewed titles and abstracts against the inclusion criteria. Where a decision could not be made on the title and abstract alone, full texts were retrieved. A second researcher (MG) independently reviewed 70 % of this sample (n = 482). Discrepancies were resolved by consensus.

### Quality assessment

A single researcher completed quality checklists, including risk of bias, for each paper (NG – 48 papers; MG − 3 papers). For surveys, items adapted from Bennett et al. [26] were used, and for qualitative research, the Critical Appraisal Skills Program (CASP) checklist [27] was used.

### Data extraction

A single researcher extracted data (study characteristics, consent model, stakeholder group, and acceptability evidence) using a pre-developed data extraction tool (NG – 48 papers, MG – 3 papers).

### Analysis

Studies were categorised according to the consent model (informed, third-party, deferred, or waived) and stakeholder group (participants and their proxy decision-makers, clinical and/ or research staff and regulators). We grouped studies looking at participant views together with those looking at both participant and their proxy decision maker. Key themes related to the acceptability of each model were summarised across each sub-group [22].

## Results

### Study selection

**Fig. 1:**
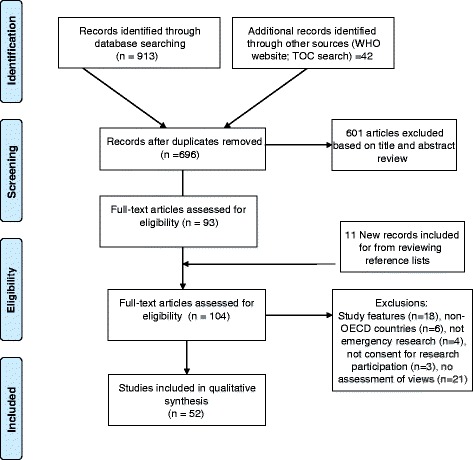
PRISMA flow diagram of the selection and inclusion of publications

We screened 695 titles and abstracts and identified 104 potentially relevant articles. Of these, 52 were excluded due to study features (n = 18), non-OECD country (n = 6), non-emergency research (n = 4), no consent for research participation (n = 3) or no assessment of views (n = 21) (Fig. 1). Our final sample included 52 papers (Tables 2, 3, 4 and 5).

**Table 2:** Characteristics of the included study – prospective informed consent

Reference	Country	Clinical context	Study design	Study aim	Scenario: real or hypothetical	Study sample		
						Respondents	Direct experience of clinical context or condition	Direct experience of consent model
Potential research participants								
Qualitative or mixed methods studies								
1. Agard 2001	Sweden	Acute myocardial infarction	Mixed methods	Investigate patient experience of consent process	Studies of early phase of treatment for myocardial infarction	31 trial participants	Yes	Yes
2. Blixen 2005^a^	USA	Stroke	Qualitative (interview)	Evaluate preferences or values	Hypothetical study – emergency stroke research	12 stroke patients	Yes	No
3. Gammelgaard 2004a	Denmark	Acute myocardial infarction	Qualitative (interviews)	Investigate patient experience of consent process	Clinical trial comparing intervention (primary angioplasty) with medical strategy (fibrinolysis)	32 trial candidates (23 participants, 9 who did not consent)	Yes	Yes
4. Mangset 2008	Norway	Stroke	Qualitative (interviews)	Investigate patient experience of consent process	Clinical trial evaluating thrombolytic drug treatment for stroke	11 trial participants	Yes	Yes
Survey studies								
5. Chenaud 2009^a^	Switzerland	ICU	Survey (Self-administered)	To evaluate preferences	Hypothetical scenarios of ICU research	67 patients; 52 relatives from recent ICU admission	Yes	No
6. Gammelgaard 2014b	Denmark	Acute myocardial infarction	Survey (Self-administered)	Investigate experience of consent process	Clinical trial comparing intervention (primary angioplasty) with medical strategy (fibrinolysis)	181 trial candidates (103 participants, 78 who did not consent)	Yes	Yes
7. Gigon 2013	Switzerland	ICU	Survey (self-administered)	Evaluate choice	Hypothetical scenarios of ICU research	185 patients, 125 relatives following ICU discharge	Yes	No
8. Paradis 2010	USA	ED research	Survey (interview)	Investigate perspectives on consent process	10 studies involving cardiac conditions	150 study participants	Yes	Yes
9. Schats 2003^a^	Netherlands	Stroke	Survey (interview)	Post-trial evaluation.	Two clinical trials that evaluated interventions for subarachnoid haemorrhage	49 patients; 47 relatives (trial participants)	Yes	Yes
10. Williams 2003	Australia, New Zealand	Acute myocardial infarction	Survey (interview)	Evaluation of consent for trial	Clinical trial of two antithrombin therapies for acute myocardial infarction	399 trial candidates	Yes	No
11. Yuval 2000	Israel	Acute myocardial infarction	Survey (Self-administered)	Post-trial evaluation	Large trial evaluating therapies for acute myocardial infarction	129 trial participants	Yes	Yes
Clinical, research staff and regulators								
Qualitative or mixed methods studies								
12. Chamberlain 2009^b^	USA	Paediatrics –status epilepticus	Qualitative (focus groups)	Evaluation during trial	Pharmacokinetic study evaluating lorazapam for status epilepticus	18 research staff	Yes	Yes

^a^compares different consent models

^b^paediatrics

**Table 3:** Characteristics of included studies – third-party consent

Reference	Country	Clinical context	Study design	Study aim	Scenario: real or hypothetical	Study sample		
						Respondents	Direct experience of clinical context or condition	Direct experience of consent model
Potential research participants								
Qualitative or mixed methods studies								
13. Ali 2006	UK	Stroke	Mixed methods	Inform clinical trial design.	Proposed trial evaluates the effect of routine oxygen supplementation after acute stroke	49 stroke patients, 24 carers	Yes	No
14. Blixen 2005^a^	USA	Stroke	Qualitative (interview)	Evaluate preferences or values	Hypothetical study – emergency stroke research	12 stroke patients	Yes	No
15. Koops 2002	UK	Stroke	Mixed methods	Inform study design.	Proposed study evaluates thrombolysis for acute ischaemic stroke	54 stroke patients and carers	Yes	No
Survey studies								
16. Barrett 2012	Canada	ICU	Survey (interview)	Evaluate attitude or opinion	Hypothetical scenarios of ICU research	136 surrogate decision makers of critically ill patients (adults and children)	Yes	No
17. Biros 2009^a^	USA	Status seizure –	Survey (self-administered)	Part of a public consultation prior to trial initiation.	Proposed trial evaluates pre-hospital intervention for status seizures	1901 community members	No	Some
18. Chenaud 2009^a^	Switzerland	ICU	Survey (Self-administered)	Evaluate preferences	Hypothetical scenarios of ICU research	67 patients; 52 relatives from recent ICU admission	Yes	No
19. Clark 2013	UK	Neurosurgery	Survey (Self-administered)	Part of a public consultation prior to trial initiation.	Proposed study evaluates surgical techniques	171 patients and carers in neuro-surgical clinic	No	No
20. Gigon 2013	Switzerland	ICU	Survey (self-administered)	Evaluate choice	Hypothetical scenarios of ICU research	185 patients, 125 relatives following ICU discharge	Yes	No
21. Kamarainen 2012^c^	Finland	Cardiac arrest	Survey (Self-administered)	Post-trial evaluation.	Trial evaluated pre-hospital intervention for cardiac arrest	11 patient; 17 consent providers; 13 physicians (trial participants)	Yes	Yes
22. Perner 2010	Denmark	ICU	Survey (self-administered)	Assess attitudes	Hypothetical trials and new medications	42 next-of-kin of unconscious ICU patients	Yes	No
23. Scales 2009^a^	Canada	Critical illness	Survey (interview)	Survey preferences	Hypothetical study scenarios of research during critical illness	240 survivors of critical illness	Yes	No
24. Schats 2003^a^	Netherlands	Stroke	Survey (interview)	Post-trial evaluation.	Two trials that evaluated interventions for subarachnoid haemorrhage	49 patients; 47 relatives (trial participants)	Yes	Yes
25. Stephenson 2007	Australia	Emergency care	Survey (self-administered)	Attitudes survey	Hypothetical scenarios of critical care research	185 patients	Possible	No
Clinical, research staff or regulators								
Survey studies								
26. Burns 2013	Canada	Pandemic research	Survey (self-administered)	Evaluate experiences, beliefs and practices	Hypothetical -pandemic research	168 administrative and clinical staff involved in H1N1 pandemic research	Yes	Yes
27. Cook 2008^c^	Canada, Australia, New Zealand	Critical illness	Survey (self-administered)	Evaluate experience, beliefs, and practices	Hypothetical – enrolment of critically ill children and adults	284 clinicians caring for critically ill patients	Yes	Yes
28. Duffett 2011^a^	Canada	Critical care research	Survey (self-administered)	Evaluate attitudes and beliefs	Hypothetical scenario of double-blind, placebo-controlled, RCT evaluating single dose of medication perceived by REB as minimal risk	98 ICU researchers; 52 members of hospital research ethics boards.	Yes	Possible
29. Kompanje 2005^a^	Netherlands	Traumatic brain injury	Survey (self-administered)	Evaluate opinions	Hypothetical -clinical emergency care	79 neuro-trauma clinical staff across 19 European countries	Yes	Possible

^a^compares different consent models

^b^compares different stakeholder groups

^c^paediatrics

**Table 4:** Characteristics of included studies – deferred consent

Reference	Country	Clinical context	Study design	Study aim	Scenario: real or hypothetical	Study sample		
						Respondents	Direct experience of clinical context or condition	Direct experience of consent model
Potential research participants								
Qualitative or mixed methods studies								
30. Woolfall 2014^b^	UK	Paediatric – status epilepticus	Qualitative (focus groups, interviews)	Inform study design	Proposed trial evaluating new treatment for status epilepticus	17 parents	Mixed	No
Survey studies								
31. Chenaud 2009^a^	Switzerland	ICU	Survey (Self-administered)	Evaluate preferences	Hypothetical scenarios of ICU research	67 patients; 52 relatives from recent ICU admission	Yes	No
32. Gamble 2012^b^	UK	Meningitis	Survey (self-administered)	Investigate views	Proposed trial evaluating two currently used treatments for emergency resuscitation and treatment	68 families	Yes	No
33. Gigon 2013^a^	Switzerland	ICU	Survey (self-administered)	Evaluate choice	Hypothetical scenarios of ICU research	185 patients, 125 relatives following ICU discharge	Yes	No
34. Potter 2013	Australia	ICU	Survey (self-administered)	Post-trial evaluation	Clinical trial evaluating two strategies for maintaining blood sugar in ICU	210 trial participants	Yes	Yes
35. Scales 2009^a^	Canada	Critical illness	Survey (interview)	Survey preferences	Hypothetical study scenarios of research during critical illness	240 survivors of critical illness	Yes	No
Clinical, research staff or regulators								
Survey studies								
36. Cook 2008^b^	Canada, Australia, New Zealand	Critical illness	Survey (self-administered)	Evaluate experience, beliefs, and practices	Hypothetical – enrolment of critically ill children and adults	284 clinicians caring for critically ill patients	Yes	Yes
37. Duffett 2011^a^	Canada	Critical care research	Survey (self-administered)	Evaluate attitudes and beliefs	Hypothetical scenario of double-blind, placebo-controlled, RCT evaluating single dose of medication perceived by REB as minimal risk	98 ICU researchers; 52 members of hospital research ethics boards.	Yes	Possible
38. Woolfall 2013^b^	UK	Paediatric – status epilepticus	Survey (self-administered)	Evaluate views and experiences	Hypothetical	45 clinical staff	Yes	Mixed

^a^compares different consent models

^b^paediatrics

**Table 5:** Characteristics of included studies – waived consent

Reference	Country	Clinical context	Study design	Study aim	Scenario: real or hypothetical	Study sample		
						Respondents	Direct experience of clinical context or condition	Direct experience of consent model
Potential research participants								
Qualitative or mixed methods studies								
39. Blixen 2005^a^	USA	Stroke	Qualitative (interview)	Evaluate preferences or values	Hypothetical study – emergency stroke research	12 stroke patients	Yes	No
40. Dickert 2009	USA	Cardiac arrest	Qualitative (interview)	Assess views	Hypothetical study scenarios for research emergency research	22 sudden cardiac death survivors	Yes	No
41. Kasner 2011	USA	Acute neurologic emergency research	Qualitative (focus group)	Evaluate views on community consultation	Hypothetical study	Patients with previous stroke or brain injury, their families, and people at risk for traumatic brain injury (n = 40)	Yes	No
42. Morris 2004	USA	Paediatrics	Qualitative (focus group and interview)	Public consultation	Proposed in-patient paediatric resuscitation clinical trial	23 parents from PICU of children who had been resuscitated; 33 staff	Yes	No
43. Raymond 2010	USA	Paediatric resuscitation	Mixed methods	Evaluation of public disclosure	Proposed in-patient resuscitation clinical trial	93 parents attending a PICU	Yes	No
44. Richardson 2005	USA	Cardiac arrest	Qualitative (focus group)	Explore attitudes about emergency research without consent	Clinical trial evaluating pre-hospital intervention for cardiac arrest	42 participants from community where study being conducted	No	No
45. Shah 2003	USA	Emergency	Qualitative (content analysis)	Recommendations for public disclosure	Documentation for real studies	4 studies from repository of mandatory documents	N/A	N/A
Survey studies								
46. Abboud 2006	USA	Cardiopulmonary arrest	Survey (interview)	Evaluate willingness to participate	Hypothetical scenarios – intervention resuscitation research	207 Patients attending an emergency department and a 213 geriatric clinic	Mixed	No
47. Baren 1999	USA	Paediatric	Survey (interview)	Public consultation (feasibility testing)	Hypothetical clinical trial evaluating treatment for posttraumatic seizures	227 Parents of children treated in the emergency department	Yes	No
48. Biros 2009^a^	USA	Status seizure –	Survey (self-administered)	Part of a public consultation prior to trial initiation.	Proposed trial evaluates pre-hospital intervention for status seizures	1901 community members	No	Some
49. Booth 2005	UK	Cardiac arrest	Survey (self-administered)	Assess attitudes	Hypothetical – emergency research	361 patients attending an emergency department	No	No
50. Bulger 2009^c^	USA	Resuscitation	Survey (interview)	Public consultation	Clinical trials evaluating pre-hospital interventions for cardiac arrest and traumatic injury	2418 representative sample of community	No	No
51. Dickert 2013	USA	Status epileptics	Survey (interview)	Assess experience and effect of public consultation	Clinical trial of a pre-hospital comparing pharmacological interventions for status epileptics	24 patients; 37 surrogate decision makers	Yes	Yes
52. Dickert 2014b	USA	Acute traumatic brain injury	Survey (various methods)	Survey nested in public consultation	Clinical trial evaluating progesterone for treatment of traumatic brain injury	2612 community consultation participants	No	No
53. Longfield 2008	USA	Traumatic haemorrhagic shock	Survey (self-administered)	Description of public consultation	Clinical trial evaluating a pre-hospital intervention for traumatic haemorrhagic shock	150 community meeting attendees	No	No
54. McClure 2003	USA	Resuscitation	Survey (interview)	Evaluation of views and public awareness of EFIC research	Studies conducted under waived consent (details unclear)	Convenience sample of 530 patients attending a hospital emergency department	No	No
55. Morris 2006	USA	Paediatric resuscitation	Survey (interview)	Assess feasibility of public consultation	Hypothetical scenarios of in-patient resuscitation clinical trials	91 parents attending a PICU	Yes	No
56. Nelson 2013	USA	Cardiac arrest	Survey (interview or self-administered)	Evaluation of patient opt-out experience	Clinical trial evaluating a pre-hospital intervention for cardiac arrest	46 community members who had opted out of participation in a study conducted under waived consent.	No	No
57. Ramsey 2011	USA	Emergency research	Survey (interview)	Evaluation of public consultation methods	Clinical trials conducted under waived consent (detailed unclear)–	Community where study being conducted –(baseline, n = 390; 11 months later, n = 325)	No	No
58. Scales 2009^a^	Canada	Critical illness	Survey (interview)	Survey preferences	Hypothetical study scenarios of research during critical illness	240 survivors of critical illness	Yes	No
59. Smithline 1998	USA	Emergency research	Survey (interview)	Evaluate opinions	Hypothetical study scenario of acute care research	Convenience sample of patients in an emergency department 212	No	No
60. Triner 2007	USA	Traumatic haemorrhagic shock	Survey (self-administered)	Evaluation of effectiveness of public disclosure	Clinical trial evaluating a pre-hospital intervention for traumatic haemorrhagic shock	Convenience sample of patients to emergency department 497	Mixed	No
Clinical, research staff or regulators								
Qualitative studies								
61. McClure 2007	USA	Resuscitation	Qualitative (interviews)	Evaluate experience	Hypothetical – based on experience of protocol review	10 institutional review board members	Yes	Yes
Survey studies								
62. Cook 2008^c^	Canada, Australia, New Zealand	Critical illness	Survey (self-administered)	Evaluate experience, beliefs, and practices	Hypothetical – enrolment of critically ill children and adults	284 clinicians caring for critically ill patients	Yes	Yes
63. DeIorio 2007	USA	Resuscitation	Survey (self-administered)	Understand attitudes	Hypothetical – based on experience of protocol review	69 research ethics board chairpersons	Yes	Mixed
64. Dickert 2014a	USA	Status epilepticus	Survey (self-administered)	Assess views and experience of public consultation	Clinical trial of pre-hospital intervention for status epilepticus	28 research staff	Yes	Yes
65. Duffett 2011^a,b^	Canada	Critical care research	Survey (self-administered)	Evaluate attitudes and beliefs	Hypothetical scenario of double-blind, placebo-controlled, RCT evaluating single dose of medication perceived by REB as minimal risk	98 ICU researchers; 52 members of hospital research ethics boards.	Yes	Possible
66. Kompanje 2005^a^	Netherlands	Traumatic brain injury	Survey (self-administered)	Evaluate opinions	Hypothetical -clinical emergency care	79 neuro-trauma clinical staff across 19 European countries	Yes	Possible
67. Schmidt 2009	USA	Severe traumatic injury	Survey (self-administered)	Evaluate opinions and experience of research staff	Real study of pre-hospital intervention for severe trauma	844 emergency medical technicians participating in the trial	Yes	Yes

^a^compares different consent models

^b^compares different stakeholder groups

^c^paediatrics

### Study characteristics

Our sample comprised studies using quantitative (n = 37), qualitative (n = 11), or mixed methods (n = 4). The number of participants in the studies ranged from 10 to 54 for qualitative studies and from 11 to 2,612 for survey studies. Several studies covered more than one consent model (n = 9) or considered more than one stakeholder view (n = 2).

Fewer studies considered the perspectives of clinical or research staff compared with potential research participant views, and just one study included regulator perspectives of third-party and deferred consent [28].

### Quality assessment

The quality of reporting of qualitative studies was generally high with most studies and provided a clear statement of research objective (n = 13; 93 %), appropriate use of qualitative methodology (n = 13; 93 %), and evidence of rigorous analysis (n = 9; 64 %).

The quality of reporting for survey studies was variable. The majority reported clear study objectives (n = 34; 95 %), methods of survey administration (n = 38; 100 %), and data analysis (n = 33; 86 %). While most papers gave some description of the research tool (n = 31; 82 %), just over half (n = 21; 55 %) described how the tool was developed and pretested (n = 23; 60 %). Few papers (n = 6; 16 %) described efforts to validate these tools. Limitations across most studies included unclear or limited representativeness of the sample (n = 21; 55 %), influence of non-response bias (n = 21; 55 % reported this) and unclear or limited generalizability of findings (n = 32; 84 %).

We did not exclude any studies on the basis of our quality assessment.

### Prospective informed consent

#### Potential research participants

Included studies evaluated the experience of patients who had the capacity to consent to emergency research participation, for example, myocardial infarction, stroke or general ICU research (n = 11) [29–39]. Much of this research was conducted with patients who had been approached to participate in trials, including both those who had consented and, in some cases, those who had not [32, 38, 39]. Views about the acceptability of prospective informed consent were mixed. While some participants expressed the importance of being given the opportunity to consent, saying that it was important for maintaining dignity [31, 38], others were opposed to being asked to make such a decision in the face of severe illness, with some even indicating that it was immoral [29, 38].

Even when a patient did provide consent, however, the process arguably might not have met the requirement for patients to be fully informed before doing so [29, 32, 36–38]. Evaluations of two clinical trials investigating treatments for acute myocardial infarction found that 19 % of 367 [36] to 28 % of 103 [32] research participants and 7 % of 78 [32] to 8 % of 32 [36] of non-participants read the information sheet, and a mismatch existed between the educational level required to comprehend the information sheet and that of the majority of participants in one study [36]. However, the perception of participants in other trials was that they were capable and sufficiently informed to make a decision and had enough time to do so [32, 34, 39].

#### Research staff and regulators

Researchers and clinicians highlighted similar concerns about how truly informed parents were when providing consent in paediatric emergency research [40]. High levels of parental distress and anxiety, lengthy and detailed documents, and the high-pressured clinical environment were key barriers identified to this consent process. No papers assessed the views of regulators or of researchers in adult populations in emergency research where patients were deemed to have capacity.

### Third-party consent

#### Potential research participants

Two survey studies on consent in the ICU setting reported that more than 85 % of research participants and their relatives found third-party consent to be acceptable (87 % of 240 [41] and 85 % of 137 [41]). There was a small decline in acceptability when risk increased (greater risk of complications in a placebo controlled randomised controlled trial (RCT) or participants had less time to decide (<3 hrs versus 24 hrs) [42]. Patients (n = 240) who had survived critical illness also indicated third-party consent as their preferred consent model in a low- (76 %) and higher- (81 %) risk study and where two low-risk treatments were compared (77 %). The study reporting the most negative views was a questionnaire study involving people in waiting rooms at emergency departments and intensive care units (ICUs) in Australia. In response to a hypothetical question about how they would feel about a relative providing consent for them to be involved in research in the event of a critical illness, 26 % were strongly in favour, 55 % were neutral, and 19 % were against this [43]. No consistent demographic factors associated with acceptability were noted across studies.

Members of the public consulted about study design were accepting of third-party consent and the need for alternative consent models, considering them necessary to feasibly conduct emergency research [44–47]. Patients and carers involved in the design of a low-risk (oxygen supplementation) [46] and a higher-risk (thrombolysis) [47] study saw value in the need for stroke research and for adaptations to informed consent processes that might make such research feasible. A survey conducted as part of community consultation for a trial evaluating a pre-hospital intervention for seizures found that 78 % (n = 1901) of respondents supported the concept of third-party consent, and 65 % indicated willingness to be enrolled with the consent of a family member even if there was no direct benefit to themselves [44]. A community consultation for a neurosurgical trial found 91 % (n = 171) of participants were accepting of surrogate consent by a doctor independent of the trial [45].

Two studies evaluated the experience of patients and surrogate decision makers after their involvement in research conducted using third-party consent. In a post-trial evaluation of a small pre-hospital study evaluating therapeutic hypothermia after cardiac arrest, fewer than half of patients (45 % of 11) and clinician proxy decision makers (46 % of 13) felt consent had been necessary at all under emergency research conditions, while 71 % of 17 spouses felt some form of consent was necessary [48]. Reasons for this discrepancy are not clear; however, it appears that patients and surrogate decision makers would consider deferred consent as an alternative in this context.

In a study with patient-relative pairs in ICU, most respondents wanted the patient to decide about research participation if they were able (75 % of 67 patients and 77 % of 52 relatives) when considering hypothetical scenarios [31]. In a second study, a third of patients and their relatives (31 % of 185) wanted someone other than the patient to give consent, even if the patient had capacity, particularly if the study was invasive (prospective randomised trial, with small risk and potential benefit) [33]. One conclusion reached from these studies was that patients should be given a choice about who should consent on their behalf with the option for a proxy decision maker even when patients are conscious. When asked, patients seemed to have a proxy in mind including support for a physician to act as a proxy decision maker [30, 33]. Invasiveness of a study (that is, a low-risk RCT versus observational research) did not impact preference for who should consent [33]. In a small study of patients who had experienced out-of-hospital cardiac arrest, all patient respondents (100 %, n = 11) agreed (at least to some extent) that the consent provider was able to consent on their behalf, and 88 % of spouses (n = 16) agreed that they were capable of providing consent [48]. However, the clinicians were more sceptical about spouses’ ability to make these decisions due to the emotional impact of making a decision at such a time. In other studies patients and/ or family members expressed a preference for two decision makers, particularly when a study is invasive or of higher risk, as this may alleviate the burden on the proxy decision maker [31, 33, 49].

#### Research staff and regulators

Clinical researchers endorsed third-party consent models in order to feasibly conduct critical care research; however, they had concerns about the capacity of proxy decision makers to consent on their relative’s behalf, both in a survey related to traumatic brain injury research (hypothetical) (48 %, n = 78) [50] and in a real low-risk trial of therapeutic hypothermia after cardiac arrest (61 %, n = 13) [48].

Compared with research regulators, surveyed researchers endorsed third-party consent provided by two independent physicians for a hypothetical placebo-controlled trial evaluating a single dose of medication considered to be low risk for patients with cardiac arrest when a surrogate decision maker was not available (46.4 to 54.1 % of 98 researchers versus 10.0 to 18.0 % of 52 regulators, <0.001 ([28]). However, neither group found consent by the attending interventionist involved with the trial acceptable (12.4 to 15.3 % of 98 researchers and 2 % of 52 regulators). The authors suggest that these findings may reflect the different remits of respondents: while both are concerned with the safety and integrity of research processes, researchers additionally are concerned with feasibility and timely completion of research. A survey of ICU clinical researchers considered consent by two independent physicians effective when a surrogate decision maker was not available (rated 6, IQR = 5,7 on a 7 point scale, n = 284); however, views on the ethics (4, IQR = 3,6, n = 284, and feasibility (5, IQR = 3,6, n = 284,) of this approach varied [51].

We identified just one paper specific to pandemic research [52]. In a Canadian cross-sectional survey, 74 % of 39 research coordinators and 51 % of 139 administrators with experience of conducting research during the H1N1 pandemic agreed that alternatives to third-party consent prospective were required in order to effectively recruit participants to pandemic research studies [52]. Just 14.4 % of 39 of research coordinators and 5.1 % 139 of administrators disagreed with this concept. Alternative models would include adaptations to third-party consent (for example, consent being provided by two clinicians, deferred consent, or waived consent).

### Deferred consent

#### Potential research participants

Participants in a low-risk observational study in Australia reported high levels of satisfaction with their enrolment using deferred consent [53]. The majority of these participants would have consented to participate if asked prior to enrolment (95.6 %, n = 204), reported a positive experience with their method of enrolment, were satisfied with who provided consent on their behalf (92.7 %, n = 202), and were satisfied with the decision taken on their behalf (93 %, n = 201).

Patients indicated varying degrees of acceptability to enrolment using deferred consent for hypothetical studies. A greater proportion of patients preferred a deferred consent model (77 %, n = 240) to waived consent (23 % of n = 240) in a hypothetical low-risk intervention study when they were incapacitated (in a coma and on life support) and a substitute decision maker was not present [41]. When asked to rate the acceptability of this consent model 48 % (n = 240) considered it highly acceptable and 37 % (n = 240) were neutral. However, when a substitute decision maker *was* available, participants strongly preferred a third-party consent model (76 % of n = 240). A second study evaluating the views of patient–relative pairs following ICU admission suggested acceptability of deferred consent in a non-invasive study, even if patients were conscious (59 % to 86 %, n = 185 patients; 52 % to 68 %, n = 125 relatives), with acceptability decreasing as study invasiveness increased (50 % to 60 %, n = 185 patients; 46 % to 59 % relatives) [33]. This last hypothetical scenario was of an intervention study with a 5 % risk of serious complications and necessitated daily blood tests for 5 days. Surveyed relatives of ICU patients also considered deferred consent acceptable for drug trials (69 %, n = 42), but a third of these respondents would not endorse this consent model for a new drug (28 %, n = 29) [49].

Two studies considered the acceptability of deferred consent in the design of trials in paediatric emergency research. Results of a survey with families whose child had experienced bacterial meningitis or meningococcal septicaemia indicated that the majority (67 % of 68) would be willing for their child to be enrolled under deferred consent in a trial that evaluated the effectiveness of two treatments already routinely in use for that condition (Gamble) [54]. In the event of their child’s death, 66 % of the bereaved respondents (n = 19) compared with 37 % of non-bereaved respondents (n = 49)) would have wanted to be told of their child’s enrolment at some time. In a qualitative study examining parental views on a proposed trial that aimed to evaluate an anticonvulsant not yet in standard use for paediatric seizures, participants considered deferred consent acceptable [55]. They recognised the need for this model for the feasible conduct of research, saw value in research to inform treatment for other children, and expressed trust in clinicians. The acceptability of deferred consent was also dependent on the perceived risk of the intervention. In both studies, recommendations included the need for sensitivity around timing of obtaining consent and, among bereaved parents, of the individuality of the grief process [54, 55].

#### Research staff and regulators

Clinicians perceived deferred consent as one of a number of effective strategies to promote enrolment of critically ill children and adults into clinical studies [51, 56], and the majority perceived the model as feasible and ethical [51]. Clinicians who had experienced deferred consent did not perceive an impact on their relationship with parents/family of the child (59 %, n = 27) compared with clinicians who had no experience of this model (22 %, p = 0.01), suggesting that perceptions of the model may shift with experience of using it [56]. Regulators were, however, less comfortable approving deferred consent for a hypothetical low-risk clinical trial than in approving research conducted with third-party consent and were more conservative in considering it acceptable (8 %, n = 52 participants) compared with researchers (43.3 %, n = 98 participants) [28].

### Waived consent

Most of our included studies that addressed waived consent (n = 29) were conducted in the USA (n = 25), including four that addressed paediatric research under waived consent [57–60]. These studies are governed by the Federal and Drug Administrative legislation that requires sufficient community consultation and public disclosure of this kind of research. Consequently, of the 22 studies that assessed research participant views for clinical trials with adults [30, 41, 44, 61–75] and children [57–60], more than half of these described public consultations [44, 57–59, 64, 66, 67, 69, 76] or evaluations of public disclosure [60, 72, 75]. This reflects the legislative context for this kind of research. In practice, consent is usually sought later from the patient or a surrogate decision maker, and this model may operate much like the deferred consent model described earlier.

#### Potential research participants

The acceptability of waived consent research was strongly influenced by participant beliefs and experiences, for example, with involvement in research and/or receiving medical care [70]. Several studies showed a discrepancy between the concept of waived consent and its application. For example, focus group participants expressed strong ethical objections to research conducted with waived consent, but these views shifted when discussing their personal experiences [70]. Likewise, discrepancies existed between the proportion of respondents who considered waived consent acceptable and the proportion that would be willing to participate [44, 61, 66, 67]. For example, of the attendees who took part in a public consultation for a trial evaluating pre-hospital interventions for seizures, 35.4 % of 1,901 gave support for the concept of enrolment under waived consent, whereas 51 % indicated willingness to take part [44]. However, 82 % (n = 1901) of the respondents in this study viewed it as beneficial. In a public survey for resuscitation research, 34 % (n = 530) of respondents endorsed enrolment without prior consent, whereas 70 % would be willing to participate [67]. This dropped to 49 % when the study involved a new treatment, suggesting that perceptions of risk may influence decision-making. In contrast, a higher proportion of participants in a public consultation for a trial evaluating pre-hospital intervention for severe traumatic injury were reported to ‘not object’ to the concept of enrolment without prior consent (66 % of n = 150). Of these, 82 % (n = 150) were willing for the trial to continue; however, < 67 % would want to be enrolled or be willing to enrol their family member [66]. The authors noted potential confusion, particularly among elderly attendees about the concept of waived consent for research. Taken together, these findings might suggest that while people are more conservative in accepting the concept of waived consent, possibly in a desire to protect the rights of others, they are inherently altruistic in their desire to contribute to research [44, 67].

Qualitative studies with patient populations most likely to be affected by research conducted under waived consent studies have suggested altruism and trust in the medical community as key elements of patient’s decision-making [62, 63, 65, 77]. In a small qualitative study with stroke patients, interviewees were almost unanimous (92 %, n = 11) in their endorsement of physicians consenting to their participation if a surrogate decision maker was not available [77]. Another qualitative study with sudden cardiac death survivors found that patients were more concerned about risks and benefits of study participation than with the method of consent or aspects of study design such as randomisation [62]. Some interviewees in this study were also accepting of a hypothetical study that involved some risk (1 in 10 000 risk of death) but little prospect of direct benefit. Results from a focus group study with stroke or brain injury patients and their families, as well as those at risk of such injury, suggest high levels of acceptability of research conducted under waived consent [65]. This study also highlighted confusion about key research concepts, such as equipoise and randomisation, and identified the potential for therapeutic misconception, where participants perceive they will receive better treatment by their participation in the trial. When interviewed, participants and surrogate decision makers who had been involved in a clinical trial comparing pre-hospital pharmacological interventions for status epileptics, revealed similar misunderstandings about the trial in which they had participated: 49 % of 59 respondents did not understand randomisation, whereas 25 % of 61 respondents confused research participation with long-term treatment [63]. The majority of respondents, however, endorsed their personal involvement (73 % of 61) and were favourable toward research conducted under waived consent in general (67 % of 61). Findings from these studies reveal a more nuanced picture of participant experience and perception of waived consent research.

It is a legislative requirement to consult members of the public about research conducted with waived consent in the USA, and different methods have been used to do this. Two-way communication processes, such as public meetings. were more acceptable to members of the public than one-way processes such as information via the media or posters [64, 75]. Attendance at these meeting is often low, however, and may not be representative of the community, calling into question the generalisability of their findings [44, 61, 66, 75]. Additionally, there is a need to define and target consultation efforts at the community most likely to be affected by study enrolment [58, 72]. Two surveys conducted with a convenience sample of patients attending an emergency department showed that public awareness of on-going studies conducted under a consent waiver was generally low (5 % of 530 and [67] 8 % of 497[72]). Just two of 61 participants and surrogate decision makers involved in a clinical trial comparing pre-hospital pharmacological interventions for status epileptics reported awareness of the trial prior to enrolment [63].

We identified three studies concerned with paediatric in-patient resuscitation research, all of which involved parents reported from PICU, the community most likely to be affected by this research [58–60]. Parents endorsed the need for the research to be conducted without explicit consent. They described high levels of distress and feeling overwhelmed and fearful among the reasons for not being able to take in information and provide prospective consent [59]. However, they would want to be made aware that the research was taking place and have the option to opt out. A small group of parents who would choose to opt out (15 % of 91) described the stress related to that decision, the desire for the physician to choose their child’s treatment, and not wanting to be a guinea pig as reasons for their choice [58]. A range of methods for raising awareness of active studies have been used including posters in the waiting room, brochures, and verbal explanations of the study on admission. Following this approach, the majority of parents surveyed in a PICU were aware of a paediatric resuscitation study being conducted under waived consent (81 % of 93) [60].

#### Research staff and regulators

In a European survey, waived consent was seen as acceptable for emergency traumatic brain injury research by the majority of respondents (64 %, n = 79); however, 95 % indicated that proxy consent should also be sought later [50]. Waived consent was considered effective and feasible to increase enrolment of critically ill children and adults into clinical studies; however, views on the ethical acceptability of this approach varied among clinicians and researchers across Australia and New Zealand, (n = 276) [51]. In a hypothetical low-risk RCT, regulators (4.1 %, n = 52) and researchers (22.4 % n = 98) were least comfortable approving research conducted under waived consent compared with other consent models.

In the United States, regulators experienced protocols including waived consent as more complex and time consuming to review [78, 79], with one study reporting a mean time of 8.8 h, compared with 2.3 for studies not conducted under EFIC [79]. A key challenge in applying the law involved determining what constitutes adequate community consultation and public notification [78]. Different methods, at times in combination with each other, are used to achieve this goal [76, 79]. Regulators perceived the US final rule regulation as ethically acceptable in that it protected subjects (72 %, n = 46 [78, 79] and correctly balanced this protection with the need to conduct research (69 %, n = 45 [79]). We did not identify any studies of regulator views of waived consent in Europe.

## Discussion

We reviewed publications on stakeholder acceptability of consent models for emergency research participation that might inform pandemic research preparedness. A recognition exists across all stakeholder groups that emergency research calls for a derivation of prospective informed consent that is appropriate to this context. Our findings suggest that alternative consent models are broadly acceptable to potential research participants and clinical or research staff. Less is known about regulator views; however, one study suggests they may be more conservative in approving third-party and deferred consent [28]. Our findings also highlight issues and recommendations that might enhance the acceptability of these consent models and encourage recruitment in emergency research that is likely to be applicable to future epi/pandemic research.

Critically ill patients are a particularly vulnerable population, and the ethical integrity of informed consent processes is challenging even for those who have capacity to provide consent prospectively [39]. However many studies included in our review suggest that potential research participants do understand the difficulties in conducting emergency research, support the need for it, and accept the need for alternative consent models to feasibly conduct it. Willingness to participate in research and acceptance of alternative consent models was motivated by perceived value in the importance of conducting research. Furthermore, participants appear motivated by altruism, by trust in the medical community, and, importantly, by perception of the risks and benefits to taking part [62, 80]. In many included studies, the acceptability of consent models decreased in higher risk scenarios. Issues of risk and trust are open to multiple interpretations of meaning and several qualitative studies revealed complex issues such as therapeutic misconception, where patients tend to believe they will receive superior treatment if they volunteer for a clinical trial. In a pandemic, this might be particularly salient with overestimations of the potential benefits of novel, but unproven treatments, with patients viewing research participation as a means to gain access to these treatments.

Direct experience also influenced the perception of acceptability to participants, researchers, or regulators. For example, a higher proportion of participants enrolled in a study using deferred consent found the model acceptable [53] in comparison with other studies that evaluated hypothetical scenarios [33, 41]. In addition, greater acceptability of deferred consent was observed among those paediatric clinicians who had experience of the model than those who did not [56]. Among research regulators, acceptability of waived consent has developed over time through experience of interpreting relevant legislation [78, 79]. It is important, therefore, not only to continue to evaluate the experience of these different stakeholder groups but also to ensure representation of such individuals in the development and regulatory evaluation of study protocols. Additionally, on-going research during inter-pandemic periods is needed to evaluate the way in which these models were implemented and the experience of all stakeholders in using them.

### Application to a pandemic context

Most of the included studies were conducted in emergency care but non-pandemic contexts, and the extent to which we can generalise these findings to pandemic emergency research requires investigation. Ethical acceptability is determined in part by the context in which an action occurs, and different norms might be acceptable for research conducted when a pandemic threat or impact is low compared with when it is moderate or high [19]. However, as others have argued, it is the capacity of the patient rather than the urgency of a pandemic context that determines the acceptability of using alternative consent models in research [16]. Not all acutely ill patients presenting to emergency departments will lack capacity, and findings from our review were mixed about whether potential participants preferred to consent themselves or for another to decide on their behalf. Further, these consent models are not necessarily applicable in other pandemic research contexts, such as in non-emergency situations or in primary care, where patients might be less unwell and more likely to have capacity for providing prospective informed consent. Rather, the acceptability of the consent process in all settings is judged proportionate to the likely outcome of the illness and the likely burden associated with the intervention under evaluation. For example, waived consent may be the preferred consent method for clinical trials of routinely used treatments with an established safety record, but unproven for the pandemic pathogen [20]. Findings from our review were not adequate to assess the acceptability of waived consent in such a context. Moreover, pragmatic adaptations are likely to be made. For example, in a pandemic influenza outbreak, while third-party consent might be preferable, this consent might be obtained through different communication media such as verbally, by telephone or through translators [4, 19]. Findings from our review could not capture the utility or acceptability of these pragmatic solutions.

Policy and legislative frameworks that guide the inclusion of alternatives to prospective informed consent in study protocols vary across countries and regions, impeding the ability to conduct harmonised multi-site trials. This has been a particular concern in Europe with regard to the legislative context guiding clinical trials in European Union (EU) member states and its impact on emergency research. The EU Clinical Trials Directive 2001/20/EC outlined the need for proxy consent before enrolling participants who lack capacity, with no accommodation for studies in which treatment initiation needed to occur within a narrow window of time [81]. The directive was not legally binding in all member states. Consequently, about half of the EU member states addressed this by permitting deferred consent in their national law, whereas others made no provisions for emergency research [82]. This lack of harmonization presents a barrier to setting up and conducting multi-site, clinical trials for pandemic research across Europe, as researchers must navigate the different legal requirements for obtaining consent that are ratified in national law. The European Parliament has now approved new legislation in the form of a regulation (No. 536/2014), which will be legally binding in all EU member states and will allow deferred consent for emergency research under certain circumstances [83]. This is an important step for those wishing to set up pandemic research infrastructures across Europe, where the need for a coordinated approach is considered essential.

In addition to the need for scientific and ethical rigour, pandemic research needs to be efficient in its design feasibility and speed of set up [4, 84]. Clinical trials, for example, need to be recruiting within weeks of pandemic onset to inform care decisions within that same pandemic. The strain on hospital and ICU capacity to respond to surge demands for clinical services will escalate as the pandemic impact progresses [9, 10]. Ethically, research processes should not rely unduly on clinician time that would be best spent treating patients. Research designs aligned with clinical practice, such as comparative effectiveness research, [85] may allow efficient evaluation of routinely used treatment procedures. Adaptive platform trials, set up during inter-pandemic ‘peacetime’ might also expedite inclusion and investigation of novel treatments once an epidemic or pandemic is underway [86, 87]. A platform trial is essentially a trial infrastructure in which various interventions are evaluated within a master protocol. Interventions may be added or dropped once emerging outcome data provides a pre-specified sufficiently precise estimate of effectiveness or the lack thereof. Response-adaptive trials alter the proportion of patients randomised to various arms depending on emerging trial data, with more participants randomised to the more successful intervention. These innovative study designs have raised unique ethical issues that have been debated [88, 89], including questions about the validity of informed consent procedures. Adaptive trials, for example, have been described as more complex to explain to patients, threatening patient’s ability to absorb and understand what is being asked of them [88, 90]. However response-adaptive designs may go some way to address therapeutic misconceptions by narrowing the gap between what participants believe (that trial participation will improve their outcomes) and what they experience (that they will have a greater chance of being allocated to a successful intervention) [91]. Further investigation into the preferences, experiences and acceptability of consent processes for novel study designs is required.

While new study designs and alternative consent models might hold the most promise for enabling pandemic research to progress, they also attract more intensive regulatory review than more traditional designs [78, 92]. Findings from our review suggest that, while the experience of regulators has not been well evaluated, the regulators appear to be more cautious in their judgments. This is perhaps not unexpected: regulatory bodies are tasked with protecting the rights, safety and dignity of research participants, and their decisions impact public confidence and trust in science. However the views of the public, particularly among research participants with direct experience of the use of these alternative consent models, should inform regulatory decisions around acceptability. Questions arise, however, about how best to engage with members of the public so that they might contribute to these decisions in a meaningful way.

While there is still much to learn in this complex arena, it may be possible to suggest a few areas of good practice informed by previous research in this area. Recommendations might include the following: prospective informed consent in emergency research where patients have capacity should respect patient preference for verbal summary over written study information [29, 32] and the opportunity to discuss the study prior to giving consent [37]. When enrolling participants using third-party consent, study information should be provided to participants or their legal guardians after the acute phase of illness [48], decision concordance cannot be presumed [31, 33, 93–95], and involving a second decision maker, such as a treating clinician, might alleviate the burden [96] for proxy decision makers [31, 33]. Sensitivity to timing and the quality of the communication process, particularly for bereaved relatives, is required when implementing deferred consent [55]. Community consultation and engagement should use multiple methods, the majority of which should involve two-way communication [69, 75, 76]. Partnering with community members who represent target populations might enhance a study’s exposure and acceptability [69]. Strategies for ensuring awareness for on-going studies need to be developed [72] to better understand the demographics and views of people who opt-out, thereby allowing for targeted public awareness efforts [68].

Our review has also identified areas for future study. Stakeholder perceptions related specifically to consent models for pandemic-related research need evaluating. Further research on regulator experience and views is also required, particularly in the context of legislative changes across Europe. Article 35 of Regulation No. 536/2014, effective from May 2016, makes provision for obtaining informed consent in emergency situations that will be legally binding across all member states [83]. Under this regulation, deferred consent will be legally acceptable for emergency trials conducted in EU member states; however, there is a lack of research with adults who have experienced deferred consent. Furthermore, research on the unique set of challenges for implementing alternative consent models in paediatric emergency research, including the views of children or young people, is also indicated.

### Strengths and limitations

Other systematic reviews have been conducted in this area that present a thorough and detailed examination of some samples included in our review [80, 97, 98]. However, our review set out to map the breadth and direction of evidence on acceptability from multiple stakeholder perspectives and to offer guidance for further research in some key areas not identified in these other works. We developed a comprehensive search strategy that included grey literature; however, this was not exhaustive. Decisions taken to expedite our review may have introduced human error, selection bias, and language of publication bias into our sample. We were unable to assess the effect of publication bias. As appropriate to rapid review methodology, we used narrative synthesis in our analysis [22], which lacks the depth and detail of more formal methods such as meta-analysis or meta-synthesis. The heterogeneity across our sample, in context and in method, makes valid comparisons across studies complex. While most of our sample consisted of qualitative studies and surveys, there is variability in terms of the way these studies were designed, conducted, and reported [26]. For example, the way in which survey questions were framed, the variability in their aims (for example, assessing attitudes, opinions, preferences, or behaviours), the use of hypothetical scenarios, and the different modes of survey administration would all influence the results. While we assessed each survey for quality to judge the validity of findings in their own merit, we did not exclude any studies based on lower quality assessments.

## Conclusions

Alternative consent models will be needed to feasibly conduct some types of pandemic research, especially in relation to emergency situations. Potential research participants, their families, clinicians and research staff are broadly accepting of these alternative methods of obtaining consent for emergency research. The views of research regulators are less clear, but it is important for regulators to consider the views of various stakeholders in deciding on the direction of future regulation. Implementing these models requires balancing ethical principles of individual autonomy and social justice. In a pandemic, there may be a stronger imperative to more easily facilitate research that might confer significant benefit to society at large. These inherent tensions will require further research and greater public involvement in order to understand and document a full range of key stakeholder experiences in implementing these models, as well as to consider the acceptability to stakeholders in a pandemic context and to inform regulatory decision-making.

## Additional file

Additional file 1: Appendix A. Rapid review full search strategy. Description of data: record of the search strategy used for each database. (DOCX 60 kb)

## Abbreviations

EU: European Union
ICU: intensive care unit
PICU: paediatric intensive care unit
WHO: World Health Organisation
