# Supplementary material for: Key stakeholder perceptions about consent to participate in acute illness research: a rapid, systematic review to inform epi/pandemic research preparedness
Source: Trials. 2015 Dec 29;16:591. doi: 10.1186/s13063-015-1110-6 (PMC4693405; doi:10.1186/s13063-015-1110-6)
Supplement: Additional file 1: Appendix A. — Rapid review full search strategy. Description of data: record of the search strategy used for each database. (DOCX 60 kb) [file 13063_2015_1110_MOESM1_ESM.docx]

# Rapid review final search strategies for each database

###

### Medline

Database date range: 1996 to October week 5

| \|  \| **Searches** \| **Results** \| \| --- \| --- \| --- \| \| 1 \| ((consent or decision mak*) adj3 (inform* or waive* or defer* or model* or presume* or procedure* or method* or surrogate* or communit* or universal)).mp. [mp=title, abstract, original title, name of substance word, subject heading word, keyword heading word, protocol supplementary concept word, rare disease supplementary concept word, unique identifier] \| 38195 \| \| 2 \| Informed Consent/ or Presumed Consent/ or Parental Consent/ or Informed Consent By Minors/ or Third-Party Consent/ \| 21178 \| \| 3 \| 1 or 2 \| 39758 \| \| 4 \| ((consent or decision mak*) adj3 (inform* or waive* or defer* or model* or presume* or procedure* or method* or surrogate* or communit* or universal)).mp. [mp=title, abstract, original title, name of substance word, subject heading word, keyword heading word, protocol supplementary concept word, rare disease supplementary concept word, unique identifier] \| 38195 \| \| 5 \| Informed Consent/ or Presumed Consent/ or Parental Consent/ or Informed Consent By Minors/ or Third-Party Consent/ \| 21178 \| \| 6 \| 4 or 5 \| 39758 \| \| 7 \| ((emergency or critical ill* or acute* ill* or infectious or influenza) adj3 (research or stud* or trial*)).mp. [mp=title, abstract, original title, name of substance word, subject heading word, keyword heading word, protocol supplementary concept word, rare disease supplementary concept word, unique identifier] \| 6007 \| \| 8 \| Intensive Care Units/ or Critical Care/ or Intensive Care/ \| 47728 \| \| 9 \| Emergency Medicine/ or Emergency Medical Services/ \| 29087 \| \| 10 \| Epidemics/ \| 2031 \| \| 11 \| Pandemics/ \| 3245 \| \| 12 \| Influenza, Human/ \| 24752 \| \| 13 \| 7 or 8 or 9 or 10 or 11 or 12 \| 106329 \| \| 14 \| (qualitative or ethnograph* or thematic analysis or grounded theory or audio-recorded or transcribed or verbatim or ethnograph* or content analysis technique).mp. [mp=title, abstract, original title, name of substance word, subject heading word, keyword heading word, protocol supplementary concept word, rare disease supplementary concept word, unique identifier] \| 125534 \| \| 15 \| (("semi-structured" or semistructured or unstructured or informal or "in-depth" or indepth or "face-to-face" or structured or guide) adj3 (interview* or discussion* or questionnaire*)).mp. [mp=title, abstract, original title, name of substance word, subject heading word, keyword heading word, protocol supplementary concept word, rare disease supplementary concept word, unique identifier] \| 53557 \| \| 16 \| Focus groups/ or Qualitative research/ or Interviews as topic/ or Interview, Psychological/ or ((focus or discussion) adj group*1).mp. [mp=title, abstract, original title, name of substance word, subject heading word, keyword heading word, protocol supplementary concept word, rare disease supplementary concept word, unique identifier] \| 78567 \| \| 17 \| ((Questionnaire* or interview*) and (experience* or prefer* or satisf* or acceptab* or understand* or comprehen*or factor* associat* or perception* or perceive* or attitude* or view*1 or viewpoint* or standpoint* or experience* or opinion* or concern*)).mp. [mp=title, abstract, original title, name of substance word, subject heading word, keyword heading word, protocol supplementary concept word, rare disease supplementary concept word, unique identifier] \| 257671 \| \| 18 \| process evaluation.mp. \| 1141 \| \| 19 \| mixed method*1.mp. [mp=title, abstract, original title, name of substance word, subject heading word, keyword heading word, protocol supplementary concept word, rare disease supplementary concept word, unique identifier] \| 3730 \| \| 20 \| ((prefer* or satisf* or acceptab* or understand* or comprehen*) adj3 (interview* or survey* or questionnaire* or study)).mp. [mp=title, abstract, original title, name of substance word, subject heading word, keyword heading word, protocol supplementary concept word, rare disease supplementary concept word, unique identifier] \| 24791 \| \| 21 \| ((perception* or perceive* or attitude* or view*1 or viewpoint* or standpoint* or encounter* or experience* or story or stories or narrative*1 or description* or theme* or opinion* or need*1 or concerns or motivat*) adj3 (interview* or survey* or questionnaire* or study or explor* or evaluate or investigate* or analys* or collect*)).mp. [mp=title, abstract, original title, name of substance word, subject heading word, keyword heading word, protocol supplementary concept word, rare disease supplementary concept word, unique identifier] \| 82374 \| \| 22 \| (themes adj3 (identif* or analy* or review or explor* or investigat*)).mp. [mp=title, abstract, original title, name of substance word, subject heading word, keyword heading word, protocol supplementary concept word, rare disease supplementary concept word, unique identifier] \| 7054 \| \| 23 \| Personal Satisfaction/ \| 8756 \| \| 24 \| Public Opinion/ \| 10017 \| \| 25 \| 14 or 15 or 16 or 17 or 18 or 19 or 20 or 21 or 22 or 23 or 24 \| 454396 \| \| 26 \| 3 and 13 and 25 \| 203 \| \| 27 \| limit 26 to (english language and humans and yr="1996 -Current") \| 191 \| |  |
| --- | --- | --- | --- | --- | --- | --- | --- | --- | --- | --- | --- | --- | --- | --- | --- | --- | --- | --- | --- | --- | --- | --- | --- | --- | --- | --- | --- | --- | --- | --- | --- | --- | --- | --- | --- | --- | --- | --- | --- | --- | --- | --- | --- | --- | --- | --- | --- | --- | --- | --- | --- | --- | --- | --- | --- | --- | --- | --- | --- | --- | --- | --- | --- | --- | --- | --- | --- | --- | --- | --- | --- | --- | --- | --- | --- | --- | --- | --- | --- | --- | --- | --- | --- | --- | --- |

### EMBASE

Database date range: 1996 to November 05

|  | **Searches** | **Results** |
| --- | --- | --- |
| 1 | (qualitative or ethnograph* or thematic analysis or grounded theory or audio-recorded or transcribed or verbatim or ethnograph* or content analysis technique).mp. [mp=title, abstract, subject headings, heading word, drug trade name, original title, device manufacturer, drug manufacturer, device trade name, keyword] | 188307 |
| 2 | (("semi-structured" or semistructured or unstructured or informal or "in-depth" or indepth or "face-to-face" or structured or guide) adj3 (interview* or discussion* or questionnaire*)).mp. [mp=title, abstract, subject headings, heading word, drug trade name, original title, device manufacturer, drug manufacturer, device trade name, keyword] | 85696 |
| 3 | process evaluation.mp. | 1680 |
| 4 | mixed method*1.mp. [mp=title, abstract, subject headings, heading word, drug trade name, original title, device manufacturer, drug manufacturer, device trade name, keyword] | 6004 |
| 5 | ((assoc* factor*1 or prefer* or satisf* or acceptab* or understand* or comprehen*) adj3 (interview* or survey* or questionnaire* or study)).mp. [mp=title, abstract, subject headings, heading word, drug trade name, original title, device manufacturer, drug manufacturer, device trade name, keyword] | 49257 |
| 6 | ((perception* or perceive* or attitude* or view*1 or viewpoint* or standpoint* or encounter* or experience* or story or stories or narrative*1 or description* or theme* or opinion* or need*1 or concerns or motivat*) adj3 (interview* or survey* or questionnaire* or study or explor* or evaluate or investigate* or analys* or collect*)).mp. [mp=title, abstract, subject headings, heading word, drug trade name, original title, device manufacturer, drug manufacturer, device trade name, keyword] | 171239 |
| 7 | (themes adj3 (identif* or analy* or review or explor* or investigat*)).mp. [mp=title, abstract, subject headings, heading word, drug trade name, original title, device manufacturer, drug manufacturer, device trade name, keyword] | 10655 |
| 8 | ((prefer* or satisf* or acceptab* or understand* or comprehen*) adj3 (interview* or survey* or questionnaire* or study)).mp. [mp=title, abstract, subject headings, heading word, drug trade name, original title, device manufacturer, drug manufacturer, device trade name, keyword] | 49044 |
| 9 | (focus group or ((focus or discussion) adj group*1)).mp. [mp=title, abstract, subject headings, heading word, drug trade name, original title, device manufacturer, drug manufacturer, device trade name, keyword] | 28227 |
| 10 | (qualitative research/ or semi structured interview/ or interview/ or telephone interview/ or structured interview/) and (experience* or prefer* or satisf* or acceptab* or understand* or comprehen*or factor* associat* or perception* or perceive* or attitude* or view*1 or viewpoint* or standpoint* or encounter* or experience* or story or stories or narrative*1 or theme*1 or opinion* or concerns or motivat* or need*1).mp. [mp=title, abstract, subject headings, heading word, drug trade name, original title, device manufacturer, drug manufacturer, device trade name, keyword] | 93703 |
| 11 | Public Opinion/ | 9989 |
| 12 | 1 or 2 or 3 or 4 or 5 or 6 or 7 or 8 or 9 or 10 or 11 | 469444 |
| 13 | ((consent or decision mak*) adj3 (inform* or waive* or defer* or model* or presume* or procedure* or method* or surrogate* or communit* or universal)).mp. [mp=title, abstract, subject headings, heading word, drug trade name, original title, device manufacturer, drug manufacturer, device trade name, keyword] | 96478 |
| 14 | informed consent/ | 53186 |
| 15 | 13 or 14 | 96478 |
| 16 | ((emergency or critical ill* or acute* ill* or infectious or influenza) adj3 (research or stud* or trial*)).mp. [mp=title, abstract, subject headings, heading word, drug trade name, original title, device manufacturer, drug manufacturer, device trade name, keyword] | 10765 |
| 17 | intensive care/ | 74064 |
| 18 | intensive care/ | 74064 |
| 19 | intensive care unit/ | 79611 |
| 20 | emergency care/ or emergency/ or emergency medicine/ | 66801 |
| 21 | epidemic/ | 61417 |
| 22 | pandemic/ | 7131 |
| 23 | Influenza virus A H7N1/ or Influenza virus A H7N2/ or "influenza A (H2N2)"/ or influenza C/ or influenza/ or avian influenza/ or avian influenza virus/ or Influenza virus A H3N8/ or Asian influenza/ or "Influenza B virus (B/Jing Fang/76/98)"/ or Influenza virus A H1N2/ or Hong Kong influenza/ or Influenza virus A H9N2/ or "Influenza A virus (A/Puerto Rico/8/1934(H1N1))"/ or Influenza virus A H1N1/ or Influenza virus A H5N2/ or Influenza virus A H7N7/ or Influenza virus A H3N2/ or "influenza A (H3N2)"/ or 2009 H1N1 influenza/ or Influenza virus A H7N9/ or Influenza virus A H2N2/ or influenza B/ or influenza A/ or Influenza virus B/ or Influenza virus A/ or swine influenza/ or Influenza virus A H5N1/ or pandemic influenza/ or Influenza virus/ or "influenza A (H1N1)"/ or Influenza virus A H7N3/ or Influenza virus C/ or Influenza virus A H10N7/ or swine influenza virus/ or "influenza A (H5N1)"/ | 64718 |
| 24 | 16 or 17 or 18 or 19 or 20 or 21 or 22 or 23 | 321776 |
| 25 | 12 and 15 and 24 | 609 |
| 26 | limit 25 to (human and english language and yr="1996 -Current") | 518 |
| 27 | limit 26 to article | 255 |

### PsycINFO

Database date range: 1806 to November week 1 2014

|  | **Searches** | **Results** |
| --- | --- | --- |
| 1 | ((emergency or critical ill* or acute* ill* or infectious or influenza) adj3 (research or stud* or trial*)).mp. [mp=title, abstract, heading word, table of contents, key concepts, original title, tests & measures] | 806 |
| 2 | exp Intensive Care/ | 3502 |
| 3 | exp Emergency Services/ | 5682 |
| 4 | exp Epidemics/ | 2377 |
| 5 | exp Pandemics/ | 367 |
| 6 | exp Swine Influenza/ or exp Influenza/ | 1036 |
| 7 | 1 or 2 or 3 or 4 or 5 or 6 | 12734 |
| 8 | ((consent or decision mak*) adj3 (inform* or waive* or defer* or model* or presume* or procedure* or method* or surrogate* or communit* or universal)).mp. [mp=title, abstract, heading word, table of contents, key concepts, original title, tests & measures] | 14722 |
| 9 | Informed Consent/ | 3399 |
| 10 | 8 or 9 | 14722 |
| 11 | (qualitative or ethnograph* or thematic analysis or grounded theory or audio-recorded or transcribed or verbatim or ethnograph* or content analysis technique).mp. [mp=title, abstract, heading word, table of contents, key concepts, original title, tests & measures] | 127104 |
| 12 | (("semi-structured" or semistructured or unstructured or informal or "in-depth" or indepth or "face-to-face" or structured or guide) adj3 (interview* or discussion* or questionnaire*)).mp. [mp=title, abstract, heading word, table of contents, key concepts, original title, tests & measures] | 88024 |
| 13 | ((Questionnaire* or interview*) and (experience* or prefer* or satisf* or acceptab* or understand* or comprehen*or factor* associat* or perception* or perceive* or attitude* or view*1 or viewpoint* or standpoint* or experience* or opinion* or concern*)).mp. [mp=title, abstract, heading word, table of contents, key concepts, original title, tests & measures] | 300283 |
| 14 | process evaluation.mp. | 991 |
| 15 | mixed method*1.mp. [mp=title, abstract, heading word, table of contents, key concepts, original title, tests & measures] | 9277 |
| 16 | ((prefer* or satisf* or acceptab* or understand* or comprehen*) adj3 (interview* or survey* or questionnaire* or study)).mp. [mp=title, abstract, heading word, table of contents, key concepts, original title, tests & measures] | 24119 |
| 17 | ((perception* or perceive* or attitude* or view*1 or viewpoint* or standpoint* or encounter* or experience* or story or stories or narrative*1 or description* or theme* or opinion* or need*1 or concerns or motivat*) adj3 (interview* or survey* or questionnaire* or study or explor* or evaluate or investigate* or analys* or collect*)).mp. [mp=title, abstract, heading word, table of contents, key concepts, original title, tests & measures] | 129112 |
| 18 | (themes adj3 (identif* or analy* or review or explor* or investigat*)).mp. [mp=title, abstract, heading word, table of contents, key concepts, original title, tests & measures] | 10951 |
| 19 | Qualitative research/ or Interviews/ or ((focus or discussion) adj group*1).mp. [mp=title, abstract, heading word, table of contents, key concepts, original title, tests & measures] | 34110 |
| 20 | exp Public Opinion/ | 6552 |
| 21 | 11 or 12 or 13 or 14 or 15 or 16 or 17 or 18 or 19 or 20 | 503990 |
| 22 | 7 and 10 and 21 | 56 |
| 23 | limit 22 to (human and english language and yr="1996 -Current") | 53 |

### HMIC

Database date range: none

|  | **Searches** | **Results** |
| --- | --- | --- |
| 1 | ((emergency or critical ill* or acute* ill* or infectious or influenza) adj3 (research or stud* or trial*)).mp. [mp=title, other title, abstract, heading words] | 228 |
| 2 | Intensive Care Units/ or Critical Care/ or Intensive Care/ | 1389 |
| 3 | Epidemics/ | 266 |
| 4 | Pandemics/ | 1289 |
| 5 | Emergency services/ | 555 |
| 6 | exp avian influenza/ or exp Asian influenza/ or exp swine influenza/ or exp influenza/ | 2267 |
| 7 | 1 or 2 or 3 or 4 or 5 or 6 | 4562 |
| 8 | exp Informed consent/ | 388 |
| 9 | exp Consent/ | 1216 |
| 10 | ((consent or decision mak*) adj3 (inform* or waive* or defer* or model* or presume* or procedure* or method* or surrogate* or communit* or universal)).mp. [mp=title, other title, abstract, heading words] | 1573 |
| 11 | 8 or 9 or 10 | 2258 |
| 12 | (qualitative or ethnograph* or thematic analysis or grounded theory or audio-recorded or transcribed or verbatim or ethnograph* or content analysis technique).mp. [mp=title, other title, abstract, heading words] | 9180 |
| 13 | (("semi-structured" or semistructured or unstructured or informal or "in-depth" or indepth or "face-to-face" or structured or guide) adj3 (interview* or discussion* or questionnaire*)).mp. [mp=title, other title, abstract, heading words] | 5906 |
| 14 | ((Questionnaire* or interview*) and (experience* or prefer* or satisf* or acceptab* or understand* or comprehen*or factor* associat* or perception* or perceive* or attitude* or view*1 or viewpoint* or standpoint* or experience* or opinion* or concern*)).mp. [mp=title, other title, abstract, heading words] | 19432 |
| 15 | process evaluation.mp. | 158 |
| 16 | mixed method*1.mp. [mp=title, other title, abstract, heading words] | 509 |
| 17 | ((prefer* or satisf* or acceptab* or understand* or comprehen*) adj3 (interview* or survey* or questionnaire* or study)).mp. [mp=title, other title, abstract, heading words] | 1810 |
| 18 | ((perception* or perceive* or attitude* or view*1 or viewpoint* or standpoint* or encounter* or experience* or story or stories or narrative*1 or description* or theme* or opinion* or need*1 or concerns or motivat*) adj3 (interview* or survey* or questionnaire* or study or explor* or evaluate or investigate* or analys* or collect*)).mp. [mp=title, other title, abstract, heading words] | 8595 |
| 19 | (themes adj3 (identif* or analy* or review or explor* or investigat*)).mp. [mp=title, other title, abstract, heading words] | 1057 |
| 20 | exp Patient satisfaction/ or exp Public opinion/ | 3958 |
| 21 | Focus groups/ or Qualitative research/ or Interviews/ or Surveys/ or ((focus or discussion) adj group*1).mp. [mp=title, other title, abstract, heading words] | 10819 |
| 22 | 12 or 13 or 14 or 15 or 16 or 17 or 18 or 19 or 20 or 21 | 37517 |
| 23 | 7 and 11 and 22 | 4 |
| 24 | limit 23 to yr="1996 - 2014" | 4 |

###

### Web of Science

**Indexes:**

- **Science Citation Index Expanded (SCI-EXPANDED) – 1900-present**
- **Social Sciences Citation Index (SSCI) 1956-present**

TOPIC: ((consent) AND (informed OR waive* OR defer* OR presume* OR surrogate* OR community)) *AND* TOPIC: (pandemic OR epidemic OR emergency OR acute OR infectious disease) *AND* TOPIC: (prefer* or satisf* or acceptab* or understand* or comprehen*)

Refined by: LANGUAGES: (ENGLISH ) AND WEB OF SCIENCE CATEGORIES: ( EMERGENCY MEDICINE OR MEDICINE GENERAL INTERNAL OR ETHICS OR SURGERY OR CRITICAL CARE MEDICINE OR SOCIAL SCIENCES BIOMEDICAL OR MEDICAL ETHICS OR NURSING OR SOCIAL ISSUES OR PEDIATRICS OR INFECTIOUS DISEASES OR MEDICINE LEGAL ) AND DOCUMENT TYPES: ( ARTICLE )

Timespan: 1996-2014.

## Open Grey

((consent) AND (informed OR defer* OR presume* OR community)) AND (experience* OR attitude* OR accept*) lang:"en" – n=30

Including search terms for acute or critical care limited the number of citations too far. Including an extended version of the filter for views or opinions returned no citations. The majority of citations identified through this search were unpublished theses (n=28). One document was classified as a report and another as miscellaneous. Neither of these documents met the inclusion criteria for this review.

**Cochrane Central Register of Controlled Trials**

((consent) AND (informed OR waive* OR defer* OR presume* OR surrogate* OR community)) in Title, Abstract, Keywords and (pandemic OR epidemic OR emergency OR acute OR infectious disease) in Title, Abstract, Keywords and (prefer* or satisf* or acceptab* or understand* or comprehen* or opinion*) in Title, Abstract, Keywords

Publication year from 1996

### www.who.int

((consent) AND (informed OR waive* OR defer* OR presume* OR surrogate* OR community)) AND (pandemic OR epidemic OR emergency OR acute OR infectious disease) AND (prefer* or ***satisfy**** or ***acceptable**** or understand* or ***comprehend****)
